# Special Issue: Left-Right Asymmetry and Cardiac Morphogenesis

**DOI:** 10.3390/jcdd5030040

**Published:** 2018-07-26

**Authors:** Marina Campione, Thomas Brand

**Affiliations:** 1 CNR Neuroscience Institute and Department of Biomedical Sciences, University of Padua, 35122 Padova, Italy; 2 National Heart & Lung Institute, Imperial College London, ICTEM, Hammersmith Campus, London W12 0NN, UK

The laterality of inner organs is a widespread feature in the animal kingdom. Determination of laterality requires the intricate interplay between a large variety of molecular, cellular and biophysical processes, with precise spatial and temporal control during embryogenesis [1,2,3,4,5]. Although some species-specific differences exist, most of these processes are evolutionarily conserved [6,7]. Among them, a key role is played by the Nodal signaling cascade, which is activated in most species by a cilia-driven leftward flow of extracellular fluids at the left-right organizer (LRO) in neurula stage embryos [4]. The major target and final effector of this signaling cascade is the homeobox transcription factor Pitx2, which is activated on the left side of organ primordia including the developing heart [8,9]. Any deviation from normal organ laterality can have profound health implications for the fetus or newborn. Not surprisingly, this topic has attracted the attention of both scientists and clinicians, resulting in passionate discussions, which are often based on different conceptual approaches.

This Special Issue: *Left–Right Asymmetry and Cardiac Morphogenesis* is focused on the link between left-right asymmetry and cardiac morphogenesis. It includes a series of papers describing the current state of knowledge of the mechanisms involved in left–right axis formation and the establishment of cardiac laterality and also offers a perspective on its impact on congenital heart disease (CHD).

The heart is the first organ to break symmetry in the developing embryo and onset of dextral looping is the first indication of this event [10]. Heart looping involves the primitive ventricle, the atrioventricular canal and the outflow tract, which are progressively rotated and repositioned along the dorsoventral axis. In terrestrial vertebrates (amphibians, reptiles, birds and mammals), looping is thus required to generate a chamber configuration allowing a correct alignment of pulmonary and systemic flow in the mature heart. However, looping occurs in all vertebrates, including gill-breathing fishes, and therefore existed before the separation of pulmonary and systemic circulation. Starting from this observation, Hiermeier and Männer [11] have tested the hypothesis that looping of the early embryonic heart can improve its pumping efficiency. Using a technical model of a Liebau-effect pump to simulate the valve-less embryonic heart, the authors compared pumping efficiency of a linear and a looped configuration and demonstrate that looping significantly improved pumping performance. These findings might have implications for understanding the form-function relationship of the early embryonic hearts. Looping progresses concomitantly to atrial and ventricular chamber formation. Terrestrial vertebrates have two atria, and the left and right atria are morphologically distinct and establish specific connections to the venous system on either side of the body. Noticeably, the sinoatrial region is not affected by the looping process [12], therefore its morphological asymmetry reflects left-right differences in positional identity laid down during early development. The term “cardiac laterality” includes the left and right atrial identities and dextral looping. Cardiac laterality is driven by the complex interplay of the left-right signaling pathway, in particular Pitx2 and Nodal, with regulatory genes involved in cardiac gene expression and morphogenesis [13].

The normal internal organ arrangement is defined as *situs solitus*. Developmental alterations in normal left-right patterning result in a wide spectrum of laterality phenotypes, which can be broadly classified as heterotaxy and *situs inversus*; these conditions and their anatomical and morphological characteristics have been thoroughly discussed by Anderson and colleagues [14]. *Situs inversus* is a condition in which all internal organs present a reversed left-right identity. In heterotaxy, asymmetries in structure and placement of organs occur stochastically due to failure to establish asymmetry or errors in relay of axial patterning. In particular, within the heart, the laterality of the sinoatrial region and looping directionality are not coupled. Heterotaxy also includes isomerism, a condition in which the left-right morphological differences in the sinoatrial region are absent, which is associated with the occurrence of several CHDs. Some isolated forms of CHD associated to heterotaxy can occur even in a *situs solitus* condition. Versacci et al. [15] have reviewed current data on animal models and human genetic studies and highlight that these isolated CHD might be, in some patients, the only morphological manifestation of an underlying molecular left-right patterning defect. The left identity gene *Pitx2* can also be mutated in some isolated CHD, as reviewed by Franco and colleagues [16]. Noticeably, impaired Pitx2 function has also been linked to arrhythmogenesis, thus providing a novel link between laterality and adult heart function.

The broad variety of laterality phenotypes underscores the complexity in the underlying molecular regulation, in particular, modulation of Nodal signaling. The paper by Belo et al. [17] describes the role of Cerberus-like 2 (Cerl2), an inhibitor of Nodal. As highlighted by the authors, the tight control that Cerl2 exerts on Nodal signaling is extremely important, by allowing the precise modulation of the left-right genetic program in time and space. Nodal expression and function are also the topic of the paper by Schweickert et al. [18]. Nodal expression can be inverted, bilaterally induced, or absent in embryos with an aberrant left-right axis. The authors conclude that the Nodal expression pattern may be indicative of the underlying molecular defects and propose a model to explain them: randomizations can primarily be caused by defects in the flow-generating ciliary apparatus of the LRO, whereas symmetrical pattern is the result of impaired flow sensing on the left, the right, or both sides. This original conceptual approach pinpoints functions of genes whose role in laterality determination have remained obscure. Altogether, these two papers clearly show that molecular events, which occur before and around the early neurula stage are extremely important for the establishment of the body plan. The implications of these findings are important for human teratogenicity studies, as demonstrated by T. Sadler [19]. The author proposes that the prime time for teratogenic insults, normally recognized between the third and the eighth week of embryonic development should be extended to include the first two weeks of development, the time period when the three embryonic axes are laid down.

We are very thankful to all the authors who contributed to this Special Issue: *Left–Right Asymmetry and Cardiac Morphogenesis* and we hope that their papers will contain useful information for scientists and clinicians with an interest in the fascinating topic of left-right axis formation and cardiac morphogenesis.
